# Assessment of Human Papillomavirus Vaccination in Primary Care Among Swiss University Students

**DOI:** 10.1001/jamanetworkopen.2023.3949

**Published:** 2023-03-21

**Authors:** Levy Jäger, Oliver Senn, Thomas Rosemann, Andreas Plate

**Affiliations:** 1 Institute of Primary Care, University Hospital and University of Zurich, Zurich, Switzerland

## Abstract

**Importance:**

Human papillomavirus vaccination coverage rates lie below desired thresholds in Switzerland. Because general practitioners are the main contact for the relatively rare health issues of many Swiss young adults, primary care offers an important opportunity to provide catch-up human papillomavirus vaccination.

**Objective:**

To examine the knowledge, experiences, and attitudes of Swiss university students in the context of receiving human papillomavirus vaccination during primary care visits.

**Design, Setting, and Participants:**

This self-administered, cross-sectional, web-based survey study was conducted among students of 3 universities and 1 educational institution for health professions in the Swiss Canton of Zurich. Specific questions about human papillomavirus vaccination experience were directed to respondents who had received at least 1 dose administered by a general practitioner. Responses were collected during 12-week intervals between November 11, 2020, and April 7, 2021, and data were analyzed from August 3 to August 30, 2022.

**Main Outcomes and Measures:**

The primary outcome was whether human papillomavirus vaccination had been administered on the patient’s or the general practitioner’s initiative.

**Results:**

The responses of 5524 participants (median [IQR] age, 23 [21-25] years; 3878 women [70.2%]) were analyzed. The survey completion rate was 90.9% (5524 of 6076 students who consented to participate). A total of 2029 respondents (1792 women [46.2%] and 237 men [14.6%]) reported having received at least 1 human papillomavirus vaccination dose, of whom 740 (36.5%) had received at least 1 dose administered by a general practitioner. Among these, 190 respondents (25.7%) reported that vaccine administration had occurred on their request rather than on their general practitioner’s initiative. Among all respondents, 4778 (86.5%) wanted to obtain more information about human papillomavirus vaccination at a general practitioner’s office, and 2569 (55.3%) rated acute consultations in general practice as inappropriate for addressing human papillomavirus vaccination.

**Conclusions and Relevance:**

The findings of this survey study of Swiss university students suggest that primary care shows a high potential for increasing human papillomavirus vaccination coverage rates in Switzerland. However, there was room for improvement in the proactivity of general practitioners, especially with men, and in the attitudes of students toward the appropriateness of acute consultations in general practice for addressing human papillomavirus vaccination.

## Key Points

**Question:**

What are the experiences and attitudes of Swiss university students regarding receiving human papillomavirus vaccination during primary care visits?

**Findings:**

In this survey study of 5524 Swiss university students, of whom 740 had received human papillomavirus vaccination from a general practitioner, in many cases, general practitioners had administered the vaccine on the student’s, rather than their own, initiative.

**Meaning:**

These findings suggest that primary care provides an important opportunity to increase human papillomavirus vaccination rates in Switzerland, but the proactivity of general practitioners must be increased, especially with young men.

## Teaser

This survey study examines the knowledge, experiences, and attitudes of Swiss university students in the context of receiving human papillomavirus vaccination during primary care visits.

## Introduction

Vaccination against human papillomavirus (HPV) is associated with protection against HPV infection and precancerous lesions,^1^ as well as a reduction in the incidence of invasive cervical carcinoma.^2^ In Switzerland, up to 90% of HPV-related cancers are attributable to 9-valent HPV vaccine types, of which up to 19% occur in men.^3^ Accordingly, a considerable reduction in the burden of HPV-associated disease could be achieved by sufficient coverage with a 9-valent vaccine against HPV.^4^ However, rates of HPV vaccination in Switzerland lie far below a national target initially set at 80%,^5^ with only 51% of girls and 12% of boys aged 16 years having received full vaccination in 2019.^6^

HPV vaccination has been recommended by the Swiss Federal Office of Public Health for female individuals since 2007 and for male individuals since 2015 and is covered by insurance for all genders up to the age of 27 years.^5,7,8,9^ The Swiss vaccination schedule of 2022 includes HPV vaccination as a basic vaccine for female individuals aged 11 to 14 years, as a catch-up vaccine for female individuals aged 15 to 19 years, and as a recommended vaccine for female individuals aged 20 to 26 years and male individuals aged 11 to 26 years.^8^ Recommended regimens are 2 doses at age 11 to 14 years and 3 doses at age 15 to 26 years.

General practitioners (GPs) are the primary contact person for health issues of many Swiss young adults.^10^ They, therefore, provide an ideal opportunity to offer catch-up HPV vaccination to persons who have not received it in school or pediatric care. A previous study^11^ suggested that among Swiss GPs, HPV vaccination is generally well received, and potentially eligible patients are seen regularly in clinical practice. However, a major obstacle is that young adults’ reasons for encounter in primary care in Switzerland are usually acute complaints and not vaccination-related topics.^12^ In such medical consultations, the vaccination status is rarely checked, and it may be difficult for GPs to transition from the current reason for the medical encounter to the topic of HPV vaccination.^11^ On the other hand, a growing body of literature has revealed an important lack of awareness and knowledge among university students, both in Switzerland^13,14^ and abroad.^15,16,17,18,19^ However, specific knowledge on the perceptions of young adults concerning counseling and administration experiences of HPV vaccination in primary care is lacking, but it would be essential to provide GPs with specific recommendations about how to best address catch-up HPV vaccination. The aim of this survey study was, therefore, to assess the knowledge, experience, and attitudes of Swiss university students regarding receiving HPV vaccination in the context of primary care visits.

## Methods

### Study Population and Instrument

We conducted a cross-sectional, internet-based survey developed with SurveyMonkey (Momentive, Inc) at 30 faculties or departments of 3 universities and 1 educational institution for health professions in the Canton of Zurich (eTable 1 in Supplement 1). Each participating department or faculty advertised the survey to their students either via email lists or over their own online, intranet platforms. The option to participate in a lottery (to win 1 of 5 tablet computers) was used as an incentive. Data collection occurred at 12-week intervals between November 11, 2020, and April 7, 2021, from the day of the first response at each faculty or department; no reminders were sent. Duplicates were avoided by preventing users with the same internet protocol address from answering multiple times. Providing responses to all questions on 1 page was required to proceed to the respective following page. Respondents were asked for consent to collection and analysis of their anonymized response data on the opening page of the survey, where proceeding to the questionnaire was enabled by selecting a tick box. This study follows the Checklist for Reporting Results of internet E-Surveys (CHERRIES)^20^ and the Strengthening the Reporting of Observational Studies in Epidemiology (STROBE) reporting guideline^21^ cross-sectional studies. To report results of this study, we further adhere to the American Association for Public Opinion Research (AAPOR) reporting guideline. Since the questionnaire was completely anonymous, this study did not fall under the scope of the Federal Act on Research Involving Human Beings,^22^ and the local ethics committee of the Canton of Zurich waived approval.

The questionnaire contained questions on 3 main topics: knowledge about, experience of, and attitudes toward HPV vaccination in general practice. First, in the knowledge section, respondents were asked about awareness of HPV and of HPV vaccination (yes or no). Respondents who indicated awareness of HPV were further asked to estimate the lifetime risk of contracting HPV and to indicate which gender they deemed to be most commonly affected by HPV infection.

Second, the experience section started with asking respondents whether they had ever received at least 1 HPV vaccination dose (yes, no, or cannot remember) and, if applicable, about the settings where they had received HPV vaccination. If respondents indicated having received at least 1 vaccination dose administered by a GP, they were further asked about the context (in particular, whether HPV vaccination was administered on the respondent’s or the GP’s initiative).

Third, to assess attitudes toward HPV vaccination, we asked respondents whether they wanted more information on HPV at their GP’s office and to rate the appropriateness of counseling about HPV vaccination during GP encounters in the context of a check-up, of another vaccination, or of an acute illness on a 5-point Likert scale (from very inappropriate to very appropriate). We further asked whether respondents generally wanted more information about HPV vaccination and about their preferred sources of information.

Fourth, the last page of the survey asked for demographic characteristics. See eTable 2 in Supplement 1 for the formulations of the delivered survey questions.

### Statistical Analysis

We performed statistical analyses of the survey responses between August 3 and August 30, 2022. All respondents who reached and completed the last section of the questionnaire were included as participants; no further exclusion criteria were used. Descriptive summaries are presented as counts and percentages for categorical variables and as medians with IQRs for continuous variables. Tabulated results are stratified by reported gender.

Multivariable logistic regression was used to model the association of covariates collected in the survey with 1 binary outcome per section: awareness of HPV vaccination in the section on knowledge, history of at least 1 HPV vaccination dose in the section on experience, and desire for education on HPV by the GP in the section on attitudes. Respondents indicating other gender were excluded from these analyses because of their rare occurrence (Table 1), to avoid high statistical imbalance. The model covariates included gender (women or men), age (<20, 20-25, and ≥26 years); enrollment in a health care-related program, including human or dental medicine, nursing, and allied medical professions (yes or no; see details in eTable 1 in Supplement 1); health insurance model (managed care model, standard model, or unknown); and main place of residence at age 11 to 18 years (Switzerland or outside Switzerland). Results are reported in terms of adjusted odds ratios (aORs) with corresponding 95% CIs. Crude ORs with 95% CIs were determined from corresponding univariate models using the covariate of interest as the only variable. We conducted all statistical analyses with R statistical software version 4.1.0 (R Project for Statistical Computing).^23^

**Table 1.** Answers to Questions Directed to All Respondents

Respondent characteristics	Respondents, No. (%)			
	Overall (N = 5524)	Stratified by gender		
		Women (n = 3878 [70.2%])	Men (n = 1618 [29.3%])	Other (n = 28 [0.5%])
Educational program				
Health care related	1598 (28.9)	1276 (32.9)	315 (19.5)	7 (25.0)
Non–health care related	3926 (71.1)	2602 (67.1)	1303 (80.5)	21 (75.0)
Age, median (IQR), y	23.0 (21.0-25.0)	23.0 (21.0-25.0)	23.0 (21.0-25.0)	23.5 (21.0-25.0)
Main place of residence at age 11-18 y				
Switzerland	5051 (91.4)	3593 (92.7)	1432 (88.5)	26 (92.9)
Outside Switzerland	473 (8.5)	285 (7.3)	186 (11.5)	2 (7.1)
Health insurance model				
Managed care models	3536 (64.0)	2467 (63.6)	1047 (64.7)	22 (78.6)
Standard model (free choice)	1031 (18.7)	739 (19.1)	289 (17.9)	3 (10.7)
Unknown	957 (17.3)	672 (17.3)	282 (17.4)	3 (10.7)
Has ever heard about HPV	3661 (66.3)	2740 (70.7)	902 (55.7)	19 (67.9)
Knows that a vaccination against HPV is available in Switzerland	3083 (55.8)	2417 (62.3)	651 (40.2)	15 (53.6)
Has knowingly received at least 1 HPV vaccination dose	2037 (36.9)	1792 (46.2)	237 (14.6)	8 (28.6)
Does not know whether they have received HPV vaccination	1379 (25.0)	836 (21.6)	533 (32.9)	10 (35.7)
Has regular Papanicolaou smears taken	2729 (49.4)	2705 (69.8)	14 (0.9)	10 (35.7)
Has ever been confronted with the topic of HPV in the context of cancer prevention	1882 (34.1)	1599 (57.9)	274 (30.2)	9 (47.4)
GP has ever addressed HPV vaccination				
Yes	1023 (18.5)	892 (23.0)	126 (7.8)	5 (17.9)
No	3017 (54.6)	1921 (49.5)	1085 (67.1)	11 (39.3)
Do not know	1122 (20.3)	836 (21.6)	276 (17.1)	10 (35.7)
Does not have a GP	360 (6.5)	228 (5.9)	130 (8.0)	2 (7.1)

Abbreviations: GP, general practitioner; HPV, human papillomavirus.

## Results

### Study Population

Of the 6076 students who consented to participate, 5524 completed all sections of the survey (completion rate, 90.9%) and were included as respondents in the final analyses (general characteristics are summarized in Table 1). The number of students who received the survey advertisements was not available, because we had no access to the corresponding data owing to privacy reasons. Respondents were a median (IQR) age of 23 (21-25) years (full range, 17-35 years), mostly reported female gender (3878 respondents [70.2%]), had mainly lived in Switzerland at age 11 to 18 years (5051 respondents [91.4%]), mostly had a managed care health insurance model (3536 respondents [64.0%]), and were mostly not enrolled in a health care-related program (3926 respondents [71.1%]). See eTable 2 in Supplement 1 for the answer distributions of each single question.

### Knowledge

Most respondents indicated awareness of HPV (3661 respondents [66.3%]) (Table 1) and HPV vaccination (3083 respondents [55.8%]). Awareness of HPV vaccination was significantly associated with female gender (aOR, 1.89; 95% CI, 1.67-2.14) (Table 2), increasing age (aOR for 20-25 years vs <20 years, 2.25 [95% CI, 1.86-2.71]; aOR for age ≥26 years vs <20 years, 3.87 [95% CI, 3.08-4.87]), enrollment in a health care–related program (aOR, 2.30; 95% CI, 2.00-2.65), and a standard health care insurance model (aOR, 1.27; 95% CI, 1.08-1.49). Most respondents estimated the lifetime risk of HPV infection at 21% to 40% (1150 respondents [31.4%]) (Table 3) and believed that HPV infection most commonly affects women (1813 respondents [49.5%]).

**Table 2.** Results of Logistic Regression Analyses

Covariate	Answered yes to the following question, OR (95% CI)^a^					
	Have you ever heard about HPV?		Have you ever received at least 1 HPV vaccine dose?		Would you wish to be informed about HPV at your GP’s office, independently of whether you are vaccinated against HPV?	
	Crude	Adjusted	Crude	Adjusted	Crude	Adjusted
Female gender	1.91 (1.70-2.15)	1.89 (1.67-2.14)	5.01 (4.31-5.84)	4.74 (4.07-5.53)	0.97 (0.77-1.22)	0.99 (0.78-1.24)
Age (reference: <20 y)						
20-25 y	1.86 (1.57-2.22)	2.25 (1.86-2.71)	1.21 (1.01-1.45)	1.55 (1.27-1.88)	1.15 (0.82-1.58)	1.17 (0.84-1.63)
≥ 26 y	3.17 (2.58-3.92)	3.87 (3.08-4.87)	0.93 (0.76-1.15)	1.29 (1.02-1.63)	1.01 (0.69-1.45)	0.97 (0.65-1.43)
Enrolled in health care–related program	2.11 (1.85-2.41)	2.30 (2.00-2.65)	2.33 (2.07-2.62)	2.08 (1.83-2.37)	1.01 (0.81-1.27)	1.05 (0.83-1.33)
Health insurance model (reference: managed care models)						
Standard model	1.31 (1.13-1.53)	1.27 (1.08-1.49)	1.40 (1.22-1.61)	1.35 (1.16-1.56)	0.89 (0.69-1.18)	0.87 (0.67-1.14)
Unknown	0.67 (0.58-0.77)	0.81 (0.69-0.95)	0.92 (0.79-1.07)	0.95 (0.81-1.13)	1.02 (0.77-1.37)	0.99 (0.74-1.34)
Mainly lived outside Switzerland at age 11-18 y	1.49 (1.20-1.85)	1.64 (1.30-2.05)	0.68 (0.55-0.83)	0.90 (0.72-1.13)	1.65 (1.06- 2.74)	1.75 (1.08-2.83)

Abbreviations: GP, general practitioner; HPV, human papillomavirus; OR, odds ratio.

^a^ Results are reported in terms of crude and adjusted ORs with 95% CIs. The denominator population corresponds to all included respondents except those who indicated gender as other, since their rare occurrence would have led to high statistical imbalance (n = 5496; 99.5% of total).

**Table 3.** Knowledge on HPV Vaccination

Question	Respondents, No. (%)^a^			
	Overall (N = 3661)	Stratified by gender		
		Women (n = 2740 [74.8%])	Men (n = 902 [24.6%])	Other (n = 19 [0.5%])
How high is the probability of contracting HPV during the course of your life, what would you guess?				
0%-20%	965 (26.4)	688 (25.1)	270 (30.0)	7 (36.8)
21%-40%	1150 (31.4)	889 (32.5)	257 (28.6)	4 (21.1)
41%-60%	691 (18.9)	553 (20.2)	137 (15.2)	1 (5.3)
61%-80% (correct answer)^b^	586 (16.0)	432 (15.8)	148 (16.4)	6 (31.6)
81%-100%	263 (7.2)	174 (6.4)	88 (9.8)	1 (5.3)
Which of the following gender groups do you believe is most commonly affected by HPV infection?				
Women	1813 (49.5)	1428 (52.1)	378 (41.9)	7 (36.8)
Men	475 (13.0)	333 (12.2)	139 (15.4)	3 (15.8)
Both equally (correct answer)^b^	1181 (32.3)	857 (31.3)	321 (35.6)	3 (15.8)
Do not know	192 (5.2)	122 (4.5)	64 (7.1)	6 (31.6)

Abbreviation: HPV, human papillomavirus.

^a^ Questions were directed to the subset of respondents who indicated having ever heard about HPV (n = 3661; 66.3% of total).

^b^ See Baseman et al.^24^

### Experience

Among all respondents, 2029 (36.9%) indicated having received at least 1 HPV vaccination dose (1792 women [46.2%] and 237 men [14.6%]). In adjustment, a positive answer was associated with female gender (aOR, 4.74; 95% CI, 4.07-5.53), increasing age (aOR for 20-25 years vs <20 years, 1.55; 95% CI, 1.27-1.88; aOR for age ≥26 years vs <20 years, 1.29; 95% CI, 1.02-1.63), enrollment in a health care–related program (aOR, 2.08; 95% CI, 1.83-2.37), and a standard health care insurance model (aOR, 1.35; 95% CI, 1.16-1.56) (Table 2). Overall, 740 respondents (36.3% of respondents having received at least 1 HPV vaccine dose) (Table 4) indicated having received at least 1 HPV vaccination dose administered by a GP (584 women [32.6%] and 153 men [64.6%]). Among these, 190 respondents (25.7%) indicated that HPV vaccination had been administered on their own initiative (122 women [20.9%] and 67 men [43.8%]) and 255 respondents (34.5%) reported that it had been administered on their GP’s initiative (205 women [35.1%] and 49 men [32.0%]). A total of 1023 respondents (18.5%) reported that their GP had ever addressed the topic of HPV vaccination during encounters (892 women [23.0%] and 126 men [7.8%]).

**Table 4.** Experience of HPV Vaccination

Question	Respondents, No. (%)^a^			
	Overall (N = 2037)	Stratified by gender		
		Women (n = 1792 [88.0%])	Men (n = 237 [11.6%])	Other (n = 8 [0.4%])
Where did you receive the HPV vaccine? (multiple choice)				
Pediatrician	368 (18.1)	327 (18.2)	39 (16.5)	2 (25.0)
GP	740 (36.3)	584 (32.6)	153 (64.6)	3 (37.5)
Gynecologist	452 (22.2)	448 (25.0)	3 (1.3)	1 (12.5)
Hospital	25 (1.2)	20 (1.1)	5 (2.1)	0
School physician	456 (22.4)	441 (24.6)	15 (6.3)	0
Do not know	47 (2.3)	38 (2.1)	9 (3.8)	0
Other	63 (3.1)	37 (2.1)	24 (10.1)	2 (25.0)
Under what circumstances did you receive the HPV vaccine?				
I asked my GP about HPV vaccination and they offered it	190 (9.3)	122 (6.8)	67 (28.3)	1 (12.5)
My GP addressed HPV vaccination and offered it	255 (12.5)	205 (11.4)	49 (20.7)	1 (12.5)
The HPV vaccine was administered with other routine vaccines	174 (8.5)	144 (8.0)	30 (12.7)	0
Other	62 (3.0)	58 (3.2)	4 (1.7)	0
Do not know	58 (2.8)	54 (3.0)	3 (1.3)	1 (12.5)
Respondent did not get HPV vaccination from their GP	1298 (63.7)	1208 (67.4)	84 (35.4)	5 (62.5)

Abbreviations: GP, general practitioner; HPV, human papillomavirus.

^a^ Questions were directed to the subset of respondents who indicated having received at least 1 HPV vaccine dose (n = 2037; 36.9% of total).

### Attitudes

A desire for more or better information on the topic of HPV was expressed by 3925 respondents (71.1% of total). Among these, most wanted more information at schools or university (2857 respondents [72.8%]), followed by information at physicians’ offices (2636 respondents [67.2%]) and by campaigns of the Federal Office of Public Health (2282 respondents [58.1%]). Of all respondents, 4778 (86.5%) wanted to be informed about HPV at their GP’s office, regardless of whether they were vaccinated against HPV. In multivariable adjustment, only having lived outside Switzerland at age 11 to 18 years showed a significant association with a positive answer (aOR, 1.75; 95% CI, 1.08-2.83) (Table 2). Table 4 and the Figure summarize further questions regarding the preferred sources and types of consultation for information about HPV vaccination at the GP’s office. Encounters in the context of a check-up and in the context of another vaccination were rated as very appropriate or rather appropriate by 4628 respondents (83.8%) and 4436 respondents (80.3%), respectively, whereas 2596 respondents (47.0%) deemed encounters in the context of an acute illness very inappropriate or rather inappropriate for counseling about HPV vaccination.

**Figure.**
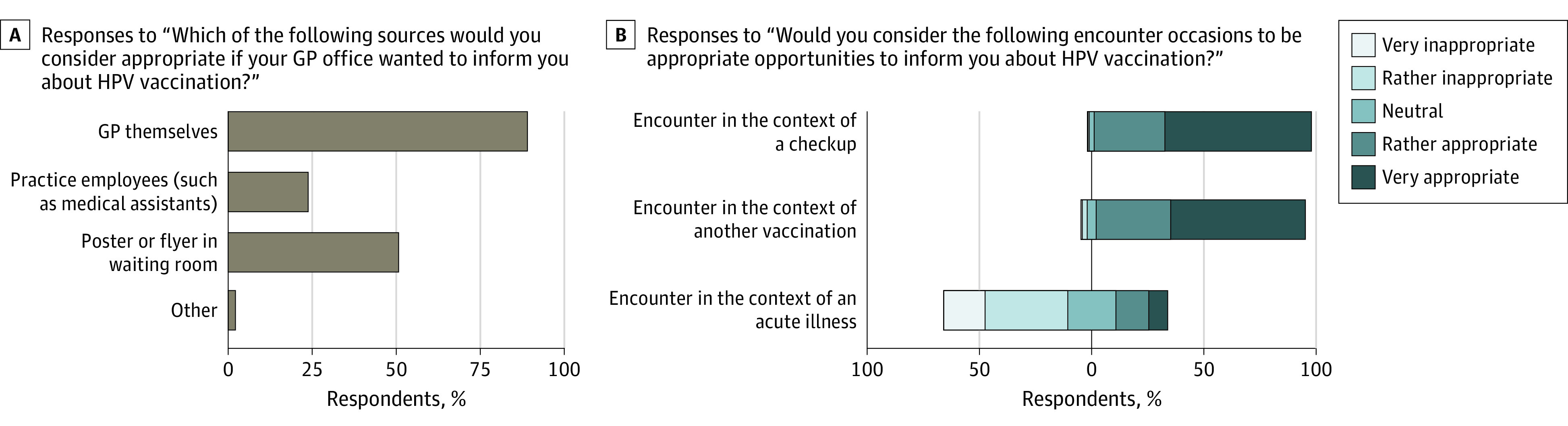
Questions Addressing Attitudes Toward Human Papillomavirus (HPV) Vaccination in General Practice A, Graph shows responses to the question, “Which of the following sources would you consider appropriate if your GP office wanted to inform you about HPV vaccination?” This question was directed to 4778 respondents who had indicated the desire for more information about HPV at their general practitioner’s (GP’s) office. B, Graph shows responses to the question, “Would you consider the following encounter occasions to be appropriate opportunities to inform you about HPV vaccination?” This question was directed to all 5524 respondents.

## Discussion

To provide evidence on how GPs could best address the topic of HPV vaccination, in this survey study, we assessed the knowledge, experience, and attitudes of Swiss university students regarding HPV vaccination in the context of primary care. In this survey with 5524 responses, we found that GPs are important providers of HPV vaccination, but that their proactivity, especially with young men, may need improvement.

### Study Population

Almost all participants reported having spent their youth in Switzerland, where HPV vaccination had been recommended for all genders for several years at the time of the survey. Accordingly, most of the study population would be expected to have been confronted with HPV vaccination at school, in pediatric care, or in gynecological care. The low HPV vaccination coverage rates we observed are similar to official national data^6^ and are in line with further estimates.^14,25^ The association of history of HPV vaccination with female gender may be linked to the fact that gynecologists are important providers of HPV vaccination in Switzerland,^26^ and the association with health care-related programs is consistent with the international literature.^19,27,28^ The association of both higher awareness of HPV and positive history of HPV vaccination with a standard health insurance model (ie, free choice of practitioner as opposed to managed-care models with gatekeeping, which provide a financial incentive by reduced premiums^29^) is, however, surprising, since coverage of HPV vaccination by health insurance is independent of the model. We may speculate that individual health care–seeking behavior could act as a confounder, influencing both the choice of the insurance model and our HPV-related outcomes.

Our results underline the need for and importance of catch-up vaccinations, because the coverage rate in our sample suggests no relevant increase compared with earlier national data for adolescents.^6^ Particularly noteworthy is the high proportion of respondents who reported not to know their vaccination status. Although this specific lack of awareness may reflect a general lack of awareness on issues related to HPV, a possible explanation lies in a decision about HPV vaccination made by the respondents’ parents during early adolescence.

### Experiences in Primary Care

GPs play an important role in the delivery of HPV vaccination. Approximately one-third of women and two-thirds of men knowingly vaccinated against HPV vaccination reported having received the vaccine from their GP and not from school health services, pediatric care, or gynecological care. The proportions found in this study are considerably higher than those reported in a national study conducted in 2015.^26^ A plausible explanation is selection bias due to our survey being spread among a section of the population pursuing higher education and due to self-selection of participants who arguably have higher awareness for health prevention measures than the general population. Still, the GP seems to be a particularly important source of HPV vaccination for men. On the other hand, the proportion of women who reported that their GP had ever addressed HPV vaccination during a consultation was more than twice as high as that of men. This may result from an at least partial focus on women in HPV counseling rather than on all genders,^11^ together with the well-known female-gendered perception among the population,^25,27^ which we were able to reproduce in our study.

Men reported more often that they had to ask their GP for HPV vaccination rather than their GP offering it on their own initiative. In addition, only 15.6% of all respondents reported that their GP had ever addressed the topic of HPV vaccination during encounters. These observations call for a change in practice.

### Addressing HPV Vaccination in Primary Care Consultations

Our study suggests that many university students want more information about HPV at their GP’s office. Interestingly, adjusted analysis revealed no association with demographic factors except for having spent adolescence outside Switzerland. This observation may reflect different perceptions of the roles of GPs as HPV vaccination providers in different health care settings.

Only about half of the respondents did not rate encounters in the context of an acute illness as inappropriate occasions to counsel about HPV. This lack of acceptance may in part explain previous observations suggesting that Swiss GPs themselves only rarely take the opportunity to offer HPV vaccination during visits for acute complaints.^11^ Still, the considerable proportion of students with neutral or positive attitudes may encourage GPs to raise the issue of HPV vaccination at every opportunity. On the other hand, our findings highlight a need for education of young patients concerning the role of GPs as providers of preventive medicine in Switzerland.

When addressing HPV, we further conclude that GPs must not assume a high level of knowledge in the target population. We observed a substantial underestimation of the lifetime risk of HPV infection, which further enhances the issue of low awareness. In particular, awareness of HPV not only affecting women cannot be taken for granted by GPs. Although respondents in health care–related educational programs showed slightly higher knowledge, we noticed large gaps even in this group. Our results are in line with both national^13,25^ and international^30,31,32,33^ data showing a large lack of knowledge in the target population, particularly among young men.

The fact that GPs had administered HPV vaccination along with other vaccines in a minority of cases highlights a further missed opportunity. The Swiss vaccination schedule recommends a diphtheria, tetanus, and pertussis booster vaccination for young adults at the age of 25 years.^8^ Although the benefit of HPV vaccination declines with increasing age of the patient,^1,2^ consultations for these routine vaccinations may still include checking the patient’s HPV vaccination status. An important opportunity, not only in the setting of primary care, might be the upcoming evidence and recommendations to adjust the HPV vaccination dosing schedule to a single-dose schedule.^34,35^ A single-dose schedule would allow vaccinating eligible patients directly on site without the need for further consultations, which would especially suit the relatively rare encounters of young adults with their GPs.

### Strengths and Limitations

A major strength of our study is the high number of respondents. Prior national studies^13,14,25^ were limited in size or recruited only in geographically confined regions. In various countries, administration of HPV vaccination relies on different health care practitioners in a similar way as in Switzerland, and the potential of providing catch-up vaccination to young adults has been emphasized in various contexts.^36,37,38,39^ Our findings are, therefore, likely to apply to other settings where GPs play an important role as the main health care providers of young adults.

This study also has limitations. Despite the high number of participants, our design does not allow accurate estimates that are generally representative of the general Swiss young adult population, especially since we restricted our sampling frame to students of higher education institutions in an urban area. Furthermore, we were not able to estimate the response rate, because we had no access to either the email lists or the login data of the intranet sites used for advertisement of the survey. In addition, potential bias may stem from self-selection of the survey respondents and recall bias, which are typical issues of web surveys.^40,41^ Arguably, the positive attitudes toward HPV vaccination may be overestimated by being associated with a general willingness to participate in an online survey on a health-related topic.

## Conclusions

GPs play an important role as providers of catch-up HPV vaccination in Switzerland. However, their proactivity needs improvement, especially with men. Most university students want more information about HPV from their GP. However, they often do not consider acute consultations in primary care to be appropriate for counseling about HPV vaccination, suggesting that there is room for improvement on their views about GPs as providers of preventive medicine.

## Notes

Supplement 1. **eTable 1.** Participating Educational Institutions **eTable 2.** Survey Questions and Response Data (n = 5524)
eReferences

Supplement 2.
Data Sharing Statement
