# Supplementary material for: Assessment of Human Papillomavirus Vaccination in Primary Care Among Swiss University Students
Source: JAMA Netw Open. 2023 Mar 21;6(3):e233949. doi: 10.1001/jamanetworkopen.2023.3949 (PMC10031396; doi:10.1001/jamanetworkopen.2023.3949)
Supplement: Supplement 1. — eTable 1. Participating Educational Institutions eTable 2. Survey Questions and Response Data (n = 5524) eReferences [file jamanetwopen-e233949-s001.pdf]

Supplemental Online Content

Jäger L, Senn O, Rosemann T, Plate A. Assessment of human papillomavirus vaccination in primary care among Swiss university students. *JAMA Netw Open*. 2023;6(3):e233949. doi:10.1001/jamanetworkopen.2023.3949

eTable 1. Participating Educational Institutions
eTable 2. Survey Questions and Response Data (n = 5524)
eReferences

This supplemental material has been provided by the authors to give readers additional information about their work.

© 2023 Jäger L et al. *JAMA Network Open.*

eTable 1. Participating Educational Institutions

Institution	Participating faculties/departments with healthcare-related programs (website)	Number of participating faculties/departments	Advertisement mode	Answering window	Number of respondents
University of Zurich	Faculty of medicine: human medicine, dental medicine, chiropractic medicine (https://www.med.uzh.ch/en.html)	7	e-mail	11/11/2020 to 03/26/2021	3489
Swiss Federal Institute of Technology Zurich	–	12	intranet/e-mail	12/01/2020 to 02/21/2021	839
Careum Bildungszentrum	Vocational college: health and social care, healthcare assistance, sterile processing technology<br>College of higher vocational education and training: nursing, biomedical laboratory diagnostics, radiology assisting, surgical assisting, dental hygiene<br>Health science: nursing<br>(https://careum.ch/en/areas/school-of-health)	3	intranet/e-mail	11/26/2020 to 03/22/2021	398
Zurich University of Applied Sciences	School of health sciences: occupational therapy, health promotion and prevention, midwifery, nursing, physiotherapy, biomedical laboratory diagnostics<br>(https://www.zhaw.ch/en/health/)	8	e-mail	02/01/2021 to 04/07/2021	1198

© 2023 Jäger L et al. *JAMA Network Open.*

eTable 2. Survey Questions and Response Data (n = 5524). Questions grouped by questionnaire section.

ID	Question, No. (%)	Overall *n* = 5524	Stratified by gender: Female *n* = 3878 (70.2%)	Male *n* = 1618 (29.3%)	Other *n* = 28 (0.5%)
	**Knowledge on HPV vaccination**				
K1	Have you ever heard about human papillomaviruses (HPV)?				
	Yes	3661 (66.3)	2740 (70.7)	902 (55.7)	19 (67.9)
	No	1863 (33.7)	1138 (29.3)	716 (44.3)	9 (32.1)
K2	In Switzerland, there is a vaccination that can safely and reliably protect against HPV infection (and thus against conditions caused by HPV). Did you know that?				
	Yes	3083 (55.8)	2417 (62.3)	651 (40.2)	15 (53.6)
	No	2441 (44.2)	1461 (37.7)	967 (59.8)	13 (46.4)
	**Questions directed to respondents who answered "Yes" to K1**				
		n = 3661	*n* = 2740 (74.8%)	*n* = 902 (24.6%)	*n* = 19 (0.5%)
K3	How high is the probability of contracting HPV during the course of your life, what would you guess?				
	0–20%	965 (26.4)	688 (25.1)	270 (29.9)	7 (36.8)
	21–40%	1150 (31.4)	889 (32.4)	257 (28.5)	4 (21.1)
	41–60%	691 (18.9)	553 (20.2)	137 (15.2)	1 (5.3)
	61–80%	586 (16.0)	432 (15.8)	148 (16.4)	6 (31.6)
	81–100%	263 (7.2)	174 (6.4)	88 (9.8)	1 (5.3)
K4	Which of the following gender groups do you believe is most commonly affected by HPV infection?				
	Women	1813 (49.5)	1428 (52.1)	378 (41.9)	7 (36.8)
	Men	475 (13.0)	333 (12.2)	139 (15.4)	3 (15.8)
	Both genders equally	1181 (32.3)	857 (31.3)	321 (35.6)	3 (15.8)
	Don't know	192 (5.2)	122 (4.5)	64 (7.1)	6 (31.6)

Abbreviations: HPV, human papillomavirus.

© 2023 Jäger L et al. *JAMA Network Open.*

eTable 2 (continued).

ID	Question, No. (%)	Overall<br>*n* = 5524	Stratified by gender<br>Female<br>*n* = 3878<br>(70.2%)	Male<br>*n* = 1618<br>(29.3%)	Other<br>*n* = 28<br>(0.5%)
	**Experience of HPV vaccination**				
E1	Do you regularly visit a gynecologist for cervical cancer screening (by means of so-called PAP smears)?				
	Yes	2729 (49.4)	2705 (69.8)	14 (0.9)	10 (35.7)
	No	1085 (19.6)	1064 (27.4)	16 (1.0)	5 (17.9)
	Don’t know	111 (2.0)	105 (2.7)	5 (0.3)	1 (3.6)
	Not applicable	1595 (28.9)	1 (0.0)	1582 (97.8)	12 (42.9)
E2	Are you vaccinated against HPV (have you received at least one vaccine dose)?				
	Yes	2037 (36.9)	1792 (46.2)	237 (14.6)	8 (28.6)
	No	2108 (38.2)	1250 (32.2)	848 (52.4)	10 (35.7)
	Can’t remember	1379 (25.0)	836 (21.6)	533 (32.9)	10 (35.7)
E3	Has your GP ever addressed HPV vaccination?				
	Yes	1023 (18.5)	892 (23.0)	126 (7.8)	5 (17.9)
	No	3017 (54.6)	1921 (49.5)	1085 (67.1)	11 (39.3)
	Don’t know	1122 (20.3)	836 (21.6)	276 (17.1)	10 (35.7)
	I don’t have a GP	360 (6.5)	228 (5.9)	130 (8.0)	2 (7.1)
E4	Have you ever asked your GP about HPV vaccination?				
	Yes	595 (10.8)	461 (11.9)	130 (8.0)	4 (14.3)
	No	4307 (78.0)	2979 (76.8)	1309 (80.9)	19 (67.9)
	Don’t know	266 (4.8)	212 (5.5)	51 (3.2)	3 (10.7)
	I don’t have a GP	356 (6.4)	226 (5.8)	128 (7.9)	2 (7.1)

Abbreviations: GP, general practitioner; HPV, human papillomavirus; PAP, Papanicolaou.

© 2023 Jäger L et al. *JAMA Network Open.*

eTable 2 (continued).

ID	Question, No. (%)	Overall	Stratified by gender		
			Female	Male	Other
		***n* = 5524**	***n* = 3878 (70.2%)**	***n* = 1618 (29.3%)**	***n* = 28 (0.5%)**
	**Question directed to respondents who answered "Yes" to E2**				
		n = 2037	*n* = 1792 (88.0%)	*n* = 237 (11.6%)	*n* = 8 (0.4%)
E5	Where did you receive the HPV vaccine? (*Multiple choice*)				
	Pediatrician	368 (18.1)	327 (18.2)	39 (16.5)	2 (25.0)
	GP	740 (36.3)	584 (32.6)	153 (64.6)	3 (37.5)
	Gynecologist	452 (22.2)	448 (25.0)	3 (1.3)	1 (12.5)
	Hospital	25 (1.2)	20 (1.1)	5 (2.1)	0 (0.0)
	School physician	456 (22.4)	441 (24.6)	15 (6.3)	0 (0.0)
	Don't know	47 (2.3)	38 (2.1)	9 (3.8)	0 (0.0)
	Other	63 (3.1)	37 (2.1)	24 (10.1)	2 (25.0)
	**Questions directed to respondents who indicated "GP" among their answers to E3**				
		n = 740	*n* = 584 (78.9%)	*n* = 153 (20.7%)	*n* = 3 (0.4%)
E6	Under what circumstances did you receive the HPV vaccine?				
	I asked my GP about HPV vaccination and they offered it	190 (25.7)	122 (20.9)	67 (43.8)	1 (33.3)
	My GP addressed HPV vaccination and offered it	255 (34.5)	205 (35.1)	49 (32.0)	1 (33.3)
	The HPV vaccine was administered with other routine vaccines	174 (23.5)	144 (24.7)	30 (19.6)	0 (0.0)
	Other	62 (8.4)	58 (9.9)	4 (2.6)	0 (0.0)
	Don't know	58 (7.8)	54 (9.2)	3 (2.0)	1 (33.3)

Abbreviations: GP, general practitioner; HPV, human papillomavirus.

© 2023 Jäger L et al. *JAMA Network Open.*

eTable 2 (continued).

ID	Question, No. (%)	Overall *n* = 5524	Female *n* = 3878 (70.2%)	Male *n* = 1618 (29.3%)	Other *n* = 28 (0.5%)
	**Attitudes towards HPV vaccination in primary care**				
A1	Would you personally like more/better information in general regarding HPV, its association with cancer, and the possibility of prevention?				
	Yes, I would like more information	3925 (71.1)	2726 (70.3)	1179 (72.9)	20 (71.4)
	No, I already feel sufficiently informed	1011 (18.3)	801 (20.7)	206 (12.7)	4 (14.3)
	No, I'd rather not address this topic	224 (4.1)	120 (3.1)	103 (6.4)	1 (3.6)
	Don't know	364 (6.6)	231 (6.0)	130 (8.0)	3 (10.7)
A2	Would you wish to be informed about HPV at your GP's office, independently of whether you are vaccinated against HPV or not?				
	Yes	4778 (86.5)	3375 (87.0)	1378 (85.2)	25 (89.3)
	No	355 (6.4)	224 (5.8)	129 (8.0)	2 (7.1)
A3	Would you consider the following encounter occasions to be appropriate opportunities to inform you about HPV vaccination? (*Likert scale with additional "No opinion" option*)				
A3-1	Encounter in the context of a check-up				
	Very inappropriate	11 (0.2)	9 (0.2)	2 (0.1)	0 (0.0)
	Rather inappropriate	24 (0.4)	20 (0.5)	4 (0.2)	0 (0.0)
	Neither inappropriate nor appropriate	105 (1.9)	79 (2.0)	25 (1.5)	1 (3.6)
	Rather appropriate	1501 (27.2)	1053 (27.2)	436 (26.9)	12 (42.9)
	Very appropriate	3127 (56.6)	2208 (56.9)	908 (56.1)	11 (39.3)
	No opinion	756 (13.4)	509 (13.1)	243 (15.0)	4 (14.3)
A3-2	Encounter in the context of another vaccination				
	Very inappropriate	24 (0.4)	17 (0.4)	7 (0.4)	0 (0.0)
	Rather inappropriate	106 (1.9)	81 (2.1)	23 (1.4)	2 (7.1)
	Neither inappropriate nor appropriate	186 (3.4)	148 (3.8)	38 (2.3)	0 (0.0)
	Rather appropriate	1585 (28.7)	1129 (29.1)	448 (27.7)	8 (28.6)
	Very appropriate	2851 (51.6)	1979 (51.0)	857 (53.0)	15 (53.6)
	No opinion	772 (14.0)	524 (13.5)	245 (15.1)	3 (10.7)
A3-3	Encounter in the context of an acute illness				
	Very inappropriate	869 (15.7)	638 (16.5)	227 (14.0)	4 (14.3)
	Rather inappropriate	1727 (31.3)	1268 (32.7)	447 (27.6)	12 (42.9)
	Neither inappropriate nor appropriate	1003 (18.2)	712 (18.4)	286 (17.7)	5 (17.9)
	Rather appropriate	692 (12.5)	426 (11.0)	263 (16.3)	3 (10.7)
	Very appropriate	403 (7.3)	269 (6.9)	133 (8.2)	1 (3.6)
	No opinion	830 (15.0)	565 (14.6)	262 (16.2)	3 (10.7)

Abbreviations: GP, general practitioner; HPV, human papillomavirus.

© 2023 Jäger L et al. *JAMA Network Open.*

eTable 2 (continued).

ID	Question, No. (%)	Overall<br>*n* = 5524	Female<br>*n* = 3878<br>(70.2%)	Male<br>*n* = 1618<br>(29.3%)	Other<br>*n* = 28<br>(0.5%)
	**Question directed to respondents who answered "Yes" to A1**				
		n = 3925	*n* = 2726<br>(69.5%)	*n* = 1179<br>(30.0%)	*n* = 20<br>(0.5%)
A4	Where would you like to obtain more information about HPV from? (*Multiple choice*)				
	Physician's office	2636 (67.2)	1952 (71.6)	669 (56.7)	15 (75.0)
	School/university/educational institutions	2857 (72.8)	2009 (73.7)	830 (70.4)	18 (90.0)
	Media	1639 (41.8)	1097 (40.2)	538 (45.6)	4 (20.0)
	Internet	1115 (28.4)	672 (24.7)	438 (37.2)	5 (25.0)
	Campaigns by the Federal Office of Public Health	2282 (58.1)	1557 (57.1)	712 (60.4)	13 (65.0)
	Other	44 (1.1)	19 (0.7)	25 (2.1)	0 (0.0)
	**Question directed to respondents who answered "Yes" to A2**				
		n = 4778	*n* = 3375<br>(70.6%)	*n* = 1378<br>(28.8%)	*n* = 25<br>(0.5%)
A5	Which of the following sources would you consider appropriate if your GP office wanted to inform you about HPV vaccination? (*Multiple choice*)				
	GP themselves	4258 (89.1)	2996 (88.8)	1242 (90.1)	20 (80.0)
	Practice employees (such as medical assistants)	1141 (23.9)	774 (22.9)	357 (25.9)	10 (40.0)
	Poster or flyer in waiting room	2426 (50.8)	1723 (51.1)	690 (50.1)	13 (52.0)
	Other	103 (2.2)	68 (2.0)	34 (2.5)	1 (4.0)
	**Demographic characteristics**				
D1	How old are you in years?	Median 23<br>(IQR 21–25)	Median 23<br>(IQR 21–25)	Median 23<br>(IQR 21–25)	Median 23.5<br>(IQR 21–25)
D2	What is your gender?				
	Female	3878 (70.2)	3878 (100)	0 (0.0)	0 (0.0)
	Male	1618 (29.3)	0 (0.0)	1618 (100)	0 (0.0)
	Other	28 (0.5)	0 (0.0)	0 (0.0)	28 (100)
D3	Which health insurance model do you have?				
	Standard model (free choice)	1031 (18.7)	739 (19.1)	289 (17.9)	3 (10.7)
	Other models (managed care)	3536 (64.0)	2467 (63.6)	1047 (64.7)	22 (78.6)
	Don't know	957 (17.3)	672 (17.3)	282 (17.4)	3 (10.7)
D4	In which department are you enrolled? (*Dropdown with the list of the educational institution's participating department*)				
	Department with healthcare-related programs only	1598 (28.9)	1276 (32.9)	315 (19.5)	7 (25.0)
	Department with no healthcare-related programs	3926 (71.1)	2602 (67.1)	1303 (80.5)	21 (75.0)
D5	Where did you mostly live between the ages of 11 and 18?				
	Switzerland	5051 (91.4)	3593 (92.7)	1432 (88.5)	26 (92.9)
	Outside Switzerland	473 (8.5)	285 (7.3)	186 (11.4)	2 (7.1)

Abbreviations: GP, general practitioner; HPV, human papillomavirus; IQR, interquartile range.

© 2023 Jäger L et al. *JAMA Network Open.*

eReferences

1. Eysenbach G. Improving the quality of Web surveys: the Checklist for Reporting Results of Internet E-Surveys (CHERRIES). *J Med Internet Res*. Sep 29 2004;6(3):e34. doi:10.2196/jmir.6.3.e34

2. Federal Act on Research Involving Human Beings of 30 September 2011 (Status as of 1 January 2020). Accessed November 20, 2022. https://www.admin.ch/opc/en/classified-compilation/20061313/index.html

3. Vandenbroucke JP, von Elm E, Altman DG, et al. Strengthening the Reporting of Observational Studies in Epidemiology (STROBE): explanation and elaboration. *PLoS Med*. Oct 16 2007;4(10):e297. doi:10.1371/journal.pmed.0040297

© 2023 Jäger L et al. *JAMA Network Open.*
