# Supplementary material for: Assessment of Human Papillomavirus Vaccination in Primary Care Among Swiss University Students
Source: JAMA Netw Open. 2023 Mar 21;6(3):e233949. doi: 10.1001/jamanetworkopen.2023.3949 (PMC10031396; doi:10.1001/jamanetworkopen.2023.3949)
Supplement: Supplement 2. — Data Sharing Statement [file jamanetwopen-e233949-s002.pdf]

Data Sharing Statement

Jäger. Assessment of Human Papillomavirus Vaccination in Primary Care Among Swiss University Students. *JAMA Netw Open*. Published March 21, 2023. doi:10.1001/jamanetworkopen.2023.3949

Data
Data available: Yes
Data types: Data (not involving human participants), Data dictionary
How to access data: The data used in the current study are available from the corresponding author Dr. Levy Jäger (levy.jaeger@usz.ch) upon reasonable request.
When available: With publication

Supporting Documents
Document types: Statistical/analytic code
How to access documents: The statistical/analytic code are available from the corresponding author Dr. Levy Jäger (levy.jaeger@usz.ch) upon reasonable request.
When available: With publication

Additional Information
Who can access the data: The data will be available to researchers whose proposed use of the data has been approved.
Types of analyses: The data will be made available for scientific research purposes.
Mechanisms of data availability: The data will be made available after approval of a proposal and with a signed data access agreement.
